# 

**Published:** 1891-06-13

**Authors:** 


### NOTICE TO CORRESPONDENTS.

All MS., Letters, Books for Review, and other matters intended for the Editor should be addressed The Editor, The Lodge, Porchester Square, London, W.

The Editor cannot undertake to return rejected M.S., even when accompanied by stamped directed envelope.

**Notice to Advertisers and Subscribers.**

All Advertisements, Orders for Copies of The Hospital, and Business Communications should be addressed to The Publisher, *not to the Editor*, The Hospital, 140, Strand, W.C.

The Cost of Subscription, post free, is as follows:—Per week, 1½d.; per quarter, 1s. 8d.; per half-year, 3s. 3d.; per annum, 6s. 6d. Foreign:—Per annum, 8s. 8d.; payable, in all cases, in advance.

**NOTICE.—The Index for Vol. IX.. (ending March, 1891) is on sale at the Office of this Journal, price 2½d., post free.**

### GENERAL CHARITIES.

[This Column is devoted to an account of work of charitable institutions other than Medical Charities. The Editor will be glad to receive reports or news of work at 140, Strand. *Philanthropy* should be plainly written on the envelope.]

The Goldsmiths' Company have given £50 to the **IRISH DISTRESSED LADIES' FUND.**

Mr. Edward Lawson will preside at the 64th anniversary festival of the **PRINTERS' PENSION CORPORATION**, which is to take place at the Whitehall rooms of the Hotel Metropole, on Friday, May 22nd. Donations will be gratefully acknowledged by Mr. J. S. Hodson, the Secretary, Gray's Inn Chambers, 20, High Holborn, W.C.

The **ROYAL CAMBRIDGE ASYLUM FOR SOLDIERS' WIDOWS** has issued its fortieth report. There are 65 widows now being benefited, but there are vacancies which can only be filled by more regiments subscribing. It is suggested that a meeting shall be held at the Mansion House to aid the Funds of this Institution. Regimental subscriptions mainly support the Asylum, the regiments having the power of voting the widows of old comrades into the Asylum. This is going more or less on the mutual system, and it is a great thing to find an Institution backed up by those for whom its benefits are intended. It seems a pity there should be voting of any kind. Surely the widow whose old age or incapacity to help herself is the greatest among the number asking admission is the one whose claim is obviously the one to be helped first.

A society which seeks practically to assist and brighten the hearts of young people who are compelled to pass what should be the happiest and brightest days of their lives in the gloom and chronic poverty of the East-end of London, will appeal to many. The gloom of the West-end of London during the fogs of the winter is not altogether a thing of joy, but if it does nothing else it may serve to bring home to the more fortunate members of society the sordid and sunless surroundings of many poor children. **THE CHILDREN'S INDUSTRIAL AND FLORICULTURAL SOCIETY**, which has its headquarters at 71, Grosvenor Street, Commercial Road, E., is, financially speaking, not a large society. It seems, however, to make up for want of size in energy and activity, and by means of its flower shows, work exhibitions, cricket club, and entertainments of various kinds, brings pleasure into the lives of many young people who from the nature of their surroundings would otherwise be likely to grow into discontented men and women and bad citizens. A penny bank and clothing club, in which an endeavour is made to induce mothers to deposit regular weekly pence for their children's clothing, has been started with some success. Mr. E. W. Hart, the Hon. Manager, asks not only for monetary assistance and gifts of flowers, books, toys, left off clothing, &c., but begs for personal help from those in the West-end who have the time to attend their shows and help to organise entertainments for the benefit of the children.

The past year of the **NATIONAL SOCIETY FOR THE PREVENTION OF CRUELTY TO CHILDREN** has shown an immense progress; its income has risen from £8,871 to £19,421. There are now sixty aid committees in England, two in Wales, and three in Ireland. Opinions of all shades have been forthcoming lately on the subject of Child Life Insurance. Papers and periodicals have denounced "the libel on the working classes." That the insured child is often victimised to obtain the insurance money is considered by many to be a phantom horror originating in the minds of hysterical people, and the abolition of child-insurance is said to strike at the spirit of thrift among the working classes. Every enthusiast naturally believes in his own cause, and this Society is convinced that in a very great number of cases gain is the sole reason for insuring a child. Like everything else, there is much to be said on both sides. Many of the healthiest, happiest children in England are insured, but it is foolish to pretend that the evidence of so many people before the House of Lords last year is all worthless. Insurance is much too easily managed, and the death of a little child far too easily accounted for; and those people who talk so glibly about the interference with people's rights, instead of reviling a Society which has done, and is doing so much, might well for a short time consider the rights of little children, for so far it is the fathers, mothers, and other natural or unnatural guardians who have had all the consideration. Many working men are upon the aid committees, and many more are amongst the subscribers, and they ought to know something about the subject of children's lives. It is better to be over-zealous than indifferent, and after all there have not been half a-dozen censures on the Society since its existence. The Society is non-sectarian, it is united, it is national, and it ought to be supported liberally. It hunts out those iniquitous haunts—baby farms (by the way, can it be true that newspaper proprietors think they have security against murderous designs in advertisers, in testimonials from ministers of religion? Can there be any so fresh and green?) and, what is still more, it comes to the rescue of the child who is made to commit a crime in order that he may be convicted, sent to an industrial school, and so taken off his parents' hands. In short, children who are submitted to any sort of cruelty are helped by the Society, whose office is 7, Harpur Street, Bloomsbury, W.C.

### SCRAPS AND GLEANINGS.

THE collection at Christ Church, Lancaster Gate, on Hospital Sunday, amounted to £1,015.

THE Duke of Cleveland has sent £5,000 to the Lord Mayor for the Hospital Sunday Fund.

THE influenza epidemic continues steadily to spread in Hamburg, and the surrounding districts.

THE Army Medical Staff held their annual dinner at the Whitehall Rooms, Hotel Metropole, on the 8th inst.

SMALL-POX has broken out in the Glasgow Sailors' Home, and five sailors and a charwoman have been removed to the hospital.

A PERFORMANCE of a living chess tournament is arranged to take place in London the first week in July in aid of the Woman's Help Society.

WE hear that Messrs. Hewett and Co., of Shadwell have given £50, and Major Crosse, of Guildford, £10 10s. to the Gorleston Cottage Hospital.

MESSRS. T. ANDROS DE LA RUE AND CO., of Bunhill Row, E C, have sent a donation of £105 to the Royal Hospital for Diseases of the Chest, City-road.

THE Salters' Company have made a grant of twenty guineas to the convalescent branch of St. John's Hospital for Diseases of the Skin, Leicester Square, W.C.

AT the 64th anniversary dinner in connection with the Licensed Victuallers' Asylum, the secretary announced donations to the amount of £10,318 as the result of the festival.

IT is reported that the members of the Leprosy Commission, who are now pursuing their researches at Simla, have found that the leprosy bacillus can be isolated and cultivated artificially.

THE weekly returns of the Registrar-General for the week ending May 30th show the births to have been 99, and the deaths 837, above the average of the corresponding weeks for the last ten years.

THE Corporation of the City of London have granted the Royal Free Hospital £252 10s., in aid of the Building Completion Fund of the Hospital, and the Grocers' Company have voted the sum of £250 for the same object.

THE first anniversary of the Sunderland Friendly Societies' Convalescent Home was marked by a large meeting on May 27th, when the benefits and necessities of the institution were brought before its friends and supporters.

A GERMAN festival will be given at the German Exhibition on Saturday, June 27th, the net receipts of which will be handed over to the committees of the German Hospital and of the German Society of Benevolence in London.

GENERAL BOOTH has offered to extend his industrial experiments to Glasgow, but there is a general feeling in that city that it will be better to await the results of the "object lesson" in London before taking any action in the matter.

ON Saturday, June 20th, a performance in aid of the funds of the University College Hospital will be given in St. George's Hall, at which a farcical comedy by Miss Adeline Votieri, entitled "Prudes and Pros," will be acted for the first time.

THE populations of the principal English towns as enumerated at the census of 1891 are now announced. That of London is now 4,211,056, an increase of 10·4 per cent. on the number in 1881. The population of Liverpool shows a decrease since 1881.

IT is expected that the Duchess of Teck will open the new convalescent home for the county of Surrey, just erected at Seaford by an anonymous benefactor, at an outlay of £25,000, including £10,000 endowment. The home will accommodate 50 patients.

THE Rev. C. H. Spurgeon, who preached last Sunday morning at the Metropolitan Tabernacle, announced that the collections for the hospitals at that place of worship would be taken next Sunday as so many of the congregation were ill. The collections at St. George's Cathedral, Southwark, were also postponed until next Sunday.

THE Great Northern Central Hospital, Holloway, has received a donation of £500 from Dr. W. F. J. Turner and James R. Upton, Esq., executors to the late Miss J. G. Harrison, being a share of the bequest left by her for charitable purposes. This sum has been added to the fund now being raised for the completion of the hospital, which is so urgently needed.

THE medical superintendent of the Fulham Board of Guardians reports that of 34 deaths that have taken place at the Fulham Infirmary during the past fortnight, half that number had succumbed to the effects of influenza, which has been very prevalent at that institution and in the workhouse. At Shepherd's Bush, Hammersmith, and Kensington, the epidemic is still reported very prevalent.

A DANCE, under the immediate patronage of the Marquis and Marchioness of Carmarthen, Lady St. Levan, Lady Agnes Townsend, and others, will take place, in aid of the London Skin Hospital, at the Westminster Town Hall, on the 25th inst. Tickets may be obtained from the Secretary, at 47, Cranbourne Street, W.C. The proceeds of the dance will be applied to meeting the heavy expenses in connection with the removal of the hospital.

ST. JOHN'S HOSPITAL FOR DISEASES OF THE SKIN, which has been incorporated under the Friendly Societies Act, held its annual meeting on May 30th. The report stated that the income for 1890 amounted to £2,969 15s. 10d., an increase of £912 18s. 2d. over that for the previous year. The expenditure for maintenance was £678 16s. 7d., and for management £163 7s. 5d., leaving a balance of £80 14s. 2d. The old debts of the institution still amount to £804.

A DRAWING-ROOM meeting of the Metropolitan Provident Medical Association was held on the 3rd inst, at the residence of Lord Brassey, who presided. The meeting was held for the purpose of advocating the claims of the association to public support. The Chairman explained that the object of the association was to induce working people to provide, by means of small periodical payments, efficient medical treatment and medicine for themselves and their families. The total receipts last year had amounted to £5,285, as against £4,580 in 1889.

### THE STUDENT OF MEDICINE.

[All communications for this Supplement should be addressed to The Editor of The Hospital, at 140, Strand, with the words, "Students' Column," written in the left hand top corner of the envelope.]

### EDITORIAL NOTES.

Cycling.—A very interesting race of cyclists recently took place on French soil from Bordeaux to Paris. From the results of the times it would appear that the French roads are superior, at least for this means of progression, to the English ones, and also that the pneumatic tyre, from a practical point of view, is not equal to the older kinds. Holbein and Mills both broke their tyres, Bates came to grief on the way down, and Edge's tyre was in a very seedy condition at the finish. It is probable, however, that with some modifications these objections may yet be overcome. The contest that was most keenly fought was between Edge, Bates, Laval, and Coullibeuf. These men rode on one machine all the way, and the greater part of the time without pace-makers. The times of arrival in Paris were as follows: (I.) Edge at 11.10 a.m.; (II.) Bates, 8 sec. later; (III.) Laval at 1.6 p.m. Thus the Englishmen beat the Frenchmen by nearly two hours. Some of the other riders—Mills, for instance—is said to have had fourteen machines on the road, and he used four.

Cambridge Honorary Degrees.—On June the 16th the University will confer the degree of Doctor of Science on some well-known men of science and medicine, among whom is Professor Flower, the Director of the British Natural History Museum, and President of the Zoological Society. Originally a student of the Middlesex and University College Hospitals, and afterwards Assistant Surgeon at the former, Professor Flower, on being appointed Curator to the Museum of the Royal College of Surgeons, gave up medicine as a profession, and devoted himself with great zeal to science, and especially that branch to which the term natural history is usually applied. Also the degree is to be conferred on Dr. Metschnikoff, the Director of the Pasteur Institute in Paris; on Sir Archibald Geikie, the Director of the British Geological Survey; and on Lord Walsingham, the well-known naturalist, on his appointment of High Steward to the University.

Criminal Photography.—At the present day much valuable work is being done on the use of photography in the detection of criminals. From some modern observations it would appear that the ear is the part that most readily reveals abnormalities of physical constitution, so often found in the criminal classes. According to some of the continental writers on this subject, there are some well-recognized types of variation, which correspond to and are possibly pathognomonic of particular forms of criminals. Photography is also coming very generally into use in our hospitals and asylums, and in some of the latter a photograph is taken of every patient admitted, and this is kept in a book together with the history of the case and the clinical notes.

Betting with Undergraduates.—Much interest has been shown in a recent case at the Oxford City Court when a certain individual, described as "commission agent," was charged with occupying rooms in Oxford for the purpose of betting. The case for the prosecution was, that the defendant had been carrying on an extensive business with undergraduates of the University, one of whom had found it better to suddenly leave the 'Varsity on account of complications. Several undergraduates gave evidence as having transacted various bets with the defendant, one of whom stated that he had not been paid his winnings.

### PRESENTATIONS.

Mr. C. E. Harold West, L.R.C.P., L.R.C.S., Edin., was presented, on resigning his house-surgeonship, with a handsome silver match-box by the matron and nurses of the institution as a mark of respect and esteem.

Dr. Buckley Pogson, on resigning the post of resident medical officer at the Durham County Hospital, was presented with a handsome clock as a wedding present by the matron and nursing staff of the institution as a mark of esteem.

### THE STUDENT OF MEDICINE—(*continued*).

### APPOINTMENTS.

At the Hospital for Consumption and Diseases of the Chest, Brompton, Sidney J. Allden, M.D., M.R.C.S., has been appointed resident medical officer. At the Blackburn and East Lancaster Infirmary, W. A. Bryant, M.B., C.M., has been appointed junior house surgeon, and W. Hartley Bunting, M.B., C.M., senior house surgeon. At the Bath Royal Mineral Water Hospital, R. Carter, M.D.Aberd., M.R.C.P.Edin., has been appointed senior physician, and Hugh Lane, L.R.C.P.Edin., M.R.C.S., honorary surgeon. At the Bradford Fever Hospital, H. Noble Joynt, M.A., M.D., Dip.State Med. Dub. Univ., has been appointed medical superintendent. At the Camberwell Provident Dispensary, Walter Alex. Atkinson, M.B., B.S.Durh., has been appointed medical officer. At the Cumberland Infirmary, Carlisle, James Brown Bird, M.B., C.M.Edin., has been appointed house surgeon. At the Hospital for Sick Children, Newcastle-on-Tyne, Walter W. Heelas, M.R.C.S., L.R.C.P., has been appointed resident medical officer. At the Manchester Royal Infirmary, Ernest Annacker, M.D. Berlin, L.R.C.P. Lond., M.R.C.S., has been appointed assistant medical officer. At the Nottingham Borough Asylum, Mapperley Hill, D. L. Davies, M.R.C.S., L.R.C.P., has been appointed assistant medical officer. At the Royal Infirmary, Newcastle-on-Tyne, Walter Ridley, M.B., M.S., F.R.C.S., has been appointed surgical registrar. At the Sussex County Hospital, Brighton, Edward Forster Maynard, M.B., C.M.Edin., has been appointed house physician. At the Torbay Hospital and Provident Dispensary, Torquay, Reginald Stephens, M.R.C.S., L.R.C.P., has been appointed junior house surgeon. At the York Dispensary, A. W. Metcalfe, M.A., M.B., B.C.Cantab., M.R.C.S., L.R.C.P., has been appointed resident medical officer.

### VACANCIES.

The following vacancies are announced this week, the amount of salary in each case being given after the name of the institution:

**Resident Medical Officers.**

Bristol City Lunatic Asylum, Second Assistant Medical Officer—Salary £120, &c.
Derbyshire General Infirmary, Resident Assistant House Surgeon.
Dorset County Asylum, Assistant Medical Officer—Salary. £120, &c.
General Infirmary at Gloucester and Gloucestershire Eye Institution, House Surgen—Salary £100, &c.
Norfolk County Asylum, Thorpe, Norwich, Temporary Assistant Medical Officer and Junior Assistant Medical Officer—Salary £109, &c.
Parish of St. Leonard, Shoreditch—Clinical Assistant for Infirmary. Salary £40, &c.
Royal Hospital for Diseases of the Chest, City Road, E.C., House Physician—Salary £40, &c.
Royal South Hants Infirmary, Southampton—Assistant to the House Surgeon.
West London Hospital, Hammersmith Road, House Physician and House Surgeon.
Wolverhampton Eye Infirmary, House Surgeon—Honorarium £25, &c.
York County Hospital, Assistant House Surgeon—Salary £40, &c.

**Non-Resident Medical Officers.**

Birmingham Dental Hospital, Honorary Dental Surgeon.
Church Local Board, Medical Officer of Health—Salary £20.
County of Aberdeen, Medical Officer for the County—Salary £400.
Guy's Hospital, five additional Assistant Dental Surgeons.
Malton Union, Medical Officer of Health—Salary £100.
Queen's College, Birmingham, Medical Tutor and Demonstrator of Anatomy, also Demonstrator of Anatomy.
Royal South London Ophthalmic Hospital, St. George's Circus, S.E., Honorary Assistant Surgeon.
Royal College of Surgeons of England, several Professors and Lecturers for the ensuing year.
Sussex County Hospital, Brighton, Surgeon and Assistant Surgeon.
University of Glasgow— Assistant Examiner in Physiology—Annual fee £30.
Victoria University College, Liverpool, Holt Professorship of Physiology—Salary £375, plus share of students' fees.

### SCHOLARSHIPS AND PRIZES.

**Bristol.**

The annual distribution of prizes of the Bristol Medical School took place on May 5th. Dr. E. Long gave out the prizes and certificates, which were awarded as follows:—

Chemistry.—Prize, A. E. H. Pinch; certificates, E. H. Marsh, F. E. Peake, J. Tilsley, F. W. Willway, W. M. Willis, F. L. Titley, E. A. Dorrell, M. C. Barber, T. W. W. Bovey, E. W. Ormerod.
Practical Chemistry.—Prize, A. E. H. Pinch; certificates, F. W. Willway, F. E. Peake, J. Tilsley, E. A. Dorrell, F. L. Titley, T. W. W. Bovey, E. W. Ormerod, E. H. Marsh, W. M. Willis, M. C. Baker.
Materia Medica and Therapeutics.—Prize, A. E. H. Pinch; certificates, J. Tilsley, E. H. Marsh, F. E. Peake.
Practical Physiology and Histology.—Prize, A. E. H. Rutherford; certificates, A. Hudson, A. B. Densham, W. Smith, A. D. Griffiths, W. T. Lydall.
Obstetric Medicine.—First certificate, G. A. Peake (disqualified for

### THE STUDENT OF MEDICINE—(*continued*).

the prize by seniority); prize, J. C. Smyth; certificates, R. Leonard, H. C. Lambert, C. M. Phillips.

Practical Surgery.—First certificate, R. C. Leonard (disqualified for the prize by seniority); prize, J. C. Smyth; certificate, G. A. Peake.

Operative Surgery.—Prize, T. M. Carter; certificate, J. Freeman.

Pathology and Morbid Anatomy.—Prize, T. M. Carter; certificates, J. Freeman, W. P. Taylor.

Practical Pathology and Morbid Anatomy.—Prize, T. M. Carter; certificate, W. P. Taylor.

Medical Jurisprudence.—Prize, J. Freeman; certificates, H. C. Lambert, A. W. Peake.

Junior Classes of Anatomy and Physiology.—Prize, A. E. H. Pinch.

Junior Class of Anatomy.—Certificates, G. B. Price, W. M. Willis, E. A. Dorrell, G. F. Gregory.

Junior Class of Physiology.—Certificates, W. M. Willis, G. F. Gregory, G. B. Price, E. A. Dorrell, D. S. Gerrish, G. J. Hare, E. W. Ormered.

Senior Class of Anatomy.—Prize, A. E. R. Rutherford; certificates, A. B. Densham, F. W: Crossman, E. H. Marsh, A. Hudson, F. W. Willway, J. Tilsley.

Senior Class of Physiology.—Prize, A. E. R. Rutherford; certificates, J. Tilsley, F. W. Willway, E. H. Marsh, F. W. Crossman, A. Hudson, F. E. Peake, A. B. Densham, C. l'O. Miall, M. C. Barber, A. D. Griffiths.

Practical Anatomy.—Prize, F. W. Willway; prosectors, certificates (equal), F. W. Crossman, F. W. Willway.

Medicine.—Prize, C. W. J. Braham; certificates, W. R. Hadwen, C. M. Phillips.

Surgery.—First certificate, A. W. Peake (disqualified for the prize by seniority); prize, J. C. Smyth; certificate, W. R. Hadwen.

Hygiene.—First certificate, J. S. Griffiths (disqualified for the prize by seniority); prize, T. M. Carter.

Gold Medal to the student who most distinguished himself during the year.—T. M. Carter.

### PASS LISTS.

#### Cambridge.

THIRD EXAMINATION FOR MEDICAL AND SURGICAL DEGREES, EASTER TERM, 1891.

Part I.—Examined and Approved.

Ds J. Attlee, Joh.; Ds G. H. A. C. Berkeley, Cai.; Ds Bradford, Cai.; Ds Cropper, Trin.; Ds Daniel, Trin.; Ds Devereux, H. Selw.; Ds Dixon, Trin.; Ds Drake, Clare; Ds Edmondson, Joh.; Ds L. G. Glover, Joh.; Ds Grimsdale, Cai.; Ds Kent, Trin.; Ds Law, Christ's; Ds Lee, H. Cav.; Ds Miller, Queens'; Ds Phear, Trin.; Ds J. J. Perkins, Emman.; Ds L. Roberts, Cai.; Ds Senior, Queens'; Ds H. Simpson, Joh.; Ds Stock, Pemb.; Ds Troutbeck, Cai.; Ds Wallace, non-collegiate; Ds Ware, Joh.; Ds Watkins, Cai.; Ds G. Wilkinson (sen.), Emman.

Part II.—Abram, B.A., Cai.; H. K. Anderson, B.A., Cai.; W. H. Beaumont, B.A., Bown.; Bennetts, B.A., Cai.; G. Calthrop, B.A., Cai.; Carruthers, B.A., Christ's; Carter, B.A., Pemb.; Colclough, B.A., Cai.; Colvin-Smith, B.A., Cai.; Crosby, B.A., Cai.; Devereux, B.A., H. Selw.; Dumbleton, M.A., Pet.; Earl, M.A., H. Cav.; Felce, B.A., Jesus; Fisher, B.A., H. Cav.; Gimson, B.A., H. Cav.; Gornall, B.A., Cath.; R. H. Hall, B.A., Pemb.; Head, Trin.; Latter, B.A., Pemb.; J. Lea, B.A., Cai.; S. Lewis, B.A., Joh.; Low, B.A., Clare; Morris, B.A., Trin.; Palmer, B.A., Down., Pethick, B.A., Down.; Phear, B.A., Trin.; Richards, B.A., Down.; Richardson, M.A., Cai.; Roberts, M.A., H. Cav.; Rowland, B.A., Cai.; Savory, B.A., H. Cav.; A. H. Smith, B.A.; King's; Surridge, B.A., Cai.; A. H. Thompson, M.A., Trin.; Watts, B.A., Joh.; West, B.A., Joh.; Wilks, B.A., Cai.

#### London—May 27.

M.B. EXAMINATION.—PASS LIST.

First Division.—Frank G. Bushnell, University College; Charles Coles, St. Bartholomew's Hospital; John N. Collins, London Hospital; Douglas R. Green and John G. Hewitson, University College; Arthur C. Lankester, St. Thomas's Hospital; Florence G. Longbottom, London School of Medicine for Women; Cyril G. Mack, St. Mary's Hospital; Llewelyn Roberts, St. Bartholomew's Hospital; William G. Rogers, Guy's Hospital; Richard G. Rows and John A. Waring, University College.

Second Division.—Albert E. Berry, Owens College and Manchester Royal Infirmary; George A. Berry, Owens College and Middlesex Hospital; Henry A. Burroughs, Liverpool Royal Infirmary; William A. Clark, St. Bartholomew's Hospital; John H. Clayton, Birmingham Medical School; Herbert V. Hickman, Guy's Hospital; Theodore H. Ionides, University College; Harold Mason, Queen's College, Queen's Hospital, and General Hospital, Birmingham; Eric L. N. Pridmore, University College; Mildred Ernestine K. Staley, London School of Medicine for Women; Walter W. H. Tate, University College; Frederic R. P. Taylor, Westminster Hospital.

#### Royal College of Surgeons of England.

The following gentlemen, having passed the necessary examinations and having conformed to the bye-laws and regulations, were at the ordinary meeting of the Council admitted members of the College:—Messrs. George Stewart Abram, L.R.C.P.Lond., student of Cambridge and University College Hospital; Alexander William Allan, L.R.C.P. Lond., of Charing Cross Hospital; John Ernest Cecil Allott, L.R.C.P. Lond., of Guy's Hospital; William Rushton Ashworth, L.R.C.P.Lond., of Westminster Hospital; Percy James Atkey, L.R.C.P.Lond., of St. Thomas's Hospital; George Walter Barber, L.R.C.P.Lond., of Middle-

### THE STUDENT OF MEDICINE—(*continued*).

sex Hospital; Frank Whinfield Bartlett, L.R.C.P.Lond., of University College Hospital; Bertram George Mortimer Baskett, L.R.C.P.Lond., of the General Hospital, Bristol; Basil Boake, L.R.C.P.Lond., of Dublin; Charles Richard Box, L.R.C.P.Lond., of St. Thomas's Hospital; William George Brett, L.R.C.P.Lond., of Guy's Hospital; Reginald Brown, L.R.C.P.Lond., of St. Bartholomew's Hospital; James Busfield, L.R.C.P.Lond., of Charing Cross Hospital; Albert Carling, L.R.C.P.Lond., of Charing Cross Hospital; Richard Clegg, L.R.C.P.Lond., of Owens College, and Royal Infirmary, Manchester; Edward Beresford Collings, L.R.C.P.Lond., of Yorkshire College and General Infirmary, Leeds; Richard Hawtrey Collins, L.R.C.P.Lond., of Charing Cross Hospital; Arthur Reginald Colyer, L.R.C.P.Lond., of Charing Cross Hospital; Thomas Edward Constant, L.R.C.P.Lond. of Charing Cross Hospital; Albert Corner, L.R.C.P. Lond., of St. Bartholomew's Hospital; Edward Cornish, L.R.C.P. Lond., of Guy's Hospital; Ernest Richard Haines Cory, L.R.C.P. Lond., of London Hospital; Cuthbert Bracey Dale, L.R.C.P.Lond., of St. Bartholomew's Hospital; Anthony Dalzell, L.R.C.P.Lond., of St. Thomas's Hospital; David Livingstone Davies, L.R.C.P.Lond., of Owens College and Royal Infirmary, Manchester; Cecil Lacy Dawson, L.R.C.P.Lond., of St. Bartholomew's Hospital; Henry Carter Castroit de Renzi, L.R.C.P.Lond., of Westminster Hospital; Charles Sempill de Segundo, L.R.C.P., of St. Bartholomew's Hospital; Appu Hennedige Charles de Silva, L.R.C.P.Lond., of Durham University; Peter Christian de Wet, L.S.A.Lond., of St Thomas's Hospital; Robert Henry Beauchamp Dudgeon, L.R.C.P.Lond., of Royal Infirmary, Liverpool; John Constable Ellis, L.R.C.P.Lond., of St. George's Hospital; George William Beaumont Featherstone, L.R.C.P.Lond., of Guy's Hospital; Ernest James Finch, L.R.C.P.Lond., of St. Mary's Hospital; Frank Chubb Ford, L.R.C.P.Lond., of St. Bartholomew's Hospital; Arthur Claude Fox, L.R.C.P.Lond., of London Hospital; John Francis, L.R.C.P.Lond., of Middlesex Hospital; Louis Arthur Francis, L.R.C.P.Lond., of St. Mary's Hospital; John Freeman, L.R.C.P.Lond., of the General Hospital, Bristol; William Frederick Gardener, L.R.C.P.Lond., of St. Thomas's Hospital; John Guest Gornall, L.R.C.P.Lond., of St. Thomas's Hospital; Geoffrey Edward Hale, L.R.C.P.Lond., of Cambridge and St. George's Hospital; John Moore Hall, M.B.R.U.I., of Queen's College and Hospital, Belfast; Denis Gratwicke Halstead, L.R.C.P.Lond., of Durham University and London Hospital; Edward Thornborough Harden, L.R.C.P.Lond., of University College Hospital; Edward Drummond Hay Hawke, L.R.C.P.Lond., of Charing Cross Hospital; Arthur Hawley, L.R.C.P.Lond., of Queen's College and Hospital, Birmingham; Alfred George Hebblethwaite, L.R.C.P.Lond., of Yorkshire College and General Infirmary, Leeds; Charles Edward Milnes Hey, L.R.C.P. Lond., of St. Mary's Hospital; Augustus Wistinghausen Hoffman, L.R.C.P. Lond., of Charing Cross Hospital; George Waddington Holton, L.R.C.P. Lond., of Owens College and Royal Infirmary, Manchester; John Armistead Home, L.R.C.P. Lond., of St. Bartholomew's Hospital; Arthur Talman Ilott, L.R.C.P. Lond., of Charing Cross Hospital; Leo Edward James, L.R.C.P. Lond., of Westminster Hospital; Alfred Jeffreys, L.R.C.P. Lond., of St. Thomas's Hospital; Frederick Johnson, L.R.C.P. Lond., of St. Bartholomew's Hospital; Henry Thomas Jones, L.R.C.P. Lond., of St. Thomas's Hospital; John Farneaux Jordan, of Queen's College and Hospital, Birmingham; Arthur Foster Keyworth, L.K.Q.C.P.I., of Owens College and Royal Infirmary, Manchester; Charles Stanley Kirton, L.R.C.P. Lond., of London Hospital; Herbert Knevitt, L.R.C.P. Lond., of London Hospital; Francis Joseph Larder, L.R.C.P. Lond., of London Hospital; Charles Ewbank Lansdown, L.R.C.P. Lond., of St. Mary's Hospital; Arnold Lawson, L.R.C.P. Lond., of Middlesex Hospital; James M'Connell, M.B.R.U.I., of Queen's College and Royal Hospital, Belfast; Francis Edward Marshall, L.R.C.P. Lond., of King's College Hospital; William Frederick Edward Milton, L.R.C.P. Lond., of St. Thomas's Hospital; William James Olivey, L.R.C.P. Lond., of St. Thomas's Hospital; Charles Sumner Patterson, L.R.C.P. Lond., of Edinburgh University and Middlesex Hospital; Charles Martin Powell, L.R.C.P. Lond., of St. Bartholomew's Hospital; Charles Porter, M.D.R.U.I., of Queen's College and North Infirmary, Cork; Arthur Edward Price, L.R.C.P. Lond., of St. Thomas's Hospital; Alfred Theodore Rake, L.R.C.P. Lond., of Guy's Hospital; James Rannie, of University of Aberdeen; Charles Kinsman Rawes, L.R.C.P. Lond., of Owens College and Royal Infirmary, Manchester; Horace Richardson, of Guy's Hospital; Alonzo George Rider, L.R.C.P. Lond., of University College Hospital; Montague Louis Bouchier Rodd, L.R.C.P. Lond., of Middlesex Hospital; William Gusterson Rogers, L.R.C.P. Lond., of Guy's Hospital; Frank Mortimer Rowland, L.R.C.P. Lond., of Cambridge University and Queen's College and Hospital, Birmingham; Arthur Rudd, L.R.C.P. Lond., of Westminster Hospital; John Harry Saunders, M.B. Melb., of Melbourne University; Leonard George Scudamore, L.R.C.P. Lond., of St. Thomas's Hospital; Bruce Gordon Seton, L.R.C.P. Lond., of St. Bartholomew's Hospital; Ernest Newlyn Smith, L.R.C.P. Lond., of University College Hospital; James Richardson Spensley, L.R.C.P. Lond., of London Hospital; James Spurr, L.S.A. Lond., of St. Mary's Hospital; William Dewing Sourrell, L.R.C.P. Lond., of Guy's Hospital; George Robert Fabris Stilwell, L.R.C.P. Lond., of St. Thomas's Hospital; Francis Edward Swinton, L.R.C.P. Lond., of St. Bartholomew's Hospital; Ernest Frank Syrett, L.R.C.P. Lond., of St. Bartholomew's Hospital; William Henry Alisoun Tebbs, of Westminster Hospital; Abraham Thomas, L.R.C.P. Lond., of Guy's Hospital; Reginald Thorpe, L.R.C.P. Lond., of St. George's Hospital; Hugh Stanley Thurston, L.R.C.P. Lond., of St. Bartholomew's Hospital; Thomas Watts, L.R.C.P. Lond., of Durham University and Royal Infirmary, Manchester; Richard Henslowe Wellington, L.R.C.P. Lond., of St. Bartholomew's Hospital; Lionel Frederick West, L.R.C.P. Lond., of Yorkshire College and General Infirmary, Leeds; Herbert Lorraine Earle Wilkes, L.R.C.P. Lond., of Guy's Hospital; and Harry Bowen Williams, L.R.C.P. Lond., of St. Thomas's Hospital.

